# *Actinotignum schaalii*: Relation to Concomitants and Connection to Patients’ Conditions in Polymicrobial Biofilms of Urinary Tract Catheters and Urines

**DOI:** 10.3390/microorganisms9030669

**Published:** 2021-03-23

**Authors:** Iva Kotásková, Vít Syrovátka, Hana Obručová, Petra Vídeňská, Barbora Zwinsová, Veronika Holá, Eva Blaštíková, Filip Růžička, Tomáš Freiberger

**Affiliations:** 1 Molecular Genetics Laboratory, Centre for Cardiovascular Surgery and Transplantation, 61600 Brno, Czech Republic; iva.kotaskova@recetox.muni.cz (I.K.); obrucova.h@gmail.com (H.O.); eva.blastikova@cktch.cz (E.B.); 2 Department of Clinical Immunology and Allergology, Medical Faculty, Masaryk University, 61600 Brno, Czech Republic; 3 Research Centre for Toxic Compounds in the Environment, Masaryk University, 61600 Brno, Czech Republic; petra.videnska@recetox.muni.cz (P.V.); barbora.zwinsova@recetox.muni.cz (B.Z.); 4 Department of Botany and Zoology, Faculty of Science, Masaryk University, 61600 Brno, Czech Republic; syrovat@sci.muni.cz; 5 Institute of Microbiology, Faculty of Medicine, St. Anne’s University Hospital, Masaryk University, 61600 Brno, Czech Republic; veronika.hola@fnusa.cz (V.H.); fruzic@fnusa.cz (F.R.)

**Keywords:** *Actinotignum*, *Actinobaculum*, hydronephrosis, urinary catheter, ureteral stent, Double-J catheter, *Propionimicrobium*, *Fusobacterium*, urobiome, microbiome, colonisation

## Abstract

*Actinotignum schaalii is* an emerging, opportunistic pathogen and its connection to non-infectious diseases and conditions, such as prostate or bladder cancer, or chronic inflammation has been proposed. Here, we analyzed 297 urine, ureteral and urinary catheter samples from 128 patients by Polymerase Chain Reaction followed by Denaturing Gradient Gel Electrophoresis and Sequencing (PCR-DGGE-S), and culture, and 29 of these samples also by *16S rRNA* Illumina sequencing, to establish *A. schaalii*’s prevalence in urinary tract-related samples, its relation to other bacteria, and its potential association with patients’ conditions and samples’ characteristics. *A. schaalii*-positive samples were significantly more diverse than *A*. *schaalii* negative and between-group diversity was higher than intra-group. *Propionimicrobium lymphophilum, Fusobacterium nucleatum, Veillonella* sp., *Morganella* sp., and *Aerococcus* sp. were significantly more often present in *A. schaalii*-positive samples; thus, we suggest these species are *A. schaalii’s* concomitants, while *Enterobacter* and Staphylococcaceae were more often identified in *A. schaalii*-negative samples; therefore, we propose *A. schaalii* and these species are mutually exclusive. Additionally, a significantly higher *A. schaalii* prevalence in patients with ureter stricture associated hydronephrosis (*p* = 0.020) was noted. We suggest that *A. schaalii* could be an early polybacterial biofilm colonizer, together with concomitant species, known for pro-inflammatory features.

## 1. Introduction

*Actinotignum schaalii* (formerly *Actinobaculum*) is a Gram-positive, rod-shaped, facultatively anaerobic, bacillus [1]. The toxin-antitoxin system and attachment pilli genes together with genes of resistance to reactive oxygen radicals [2] refer to biofilm-forming features and the ability to survive under oxidative stress—common for the inflammatory environment [2].

*Actinotignum schaalii* is typically present in the urogenital tract and has not been detected in stool [3]. It has been recognized as an emerging, opportunistic pathogen and co-agent of various, typically polymicrobial infections [1,4,5,6,7,8,9,10,11,12,13,14], easily overlooked due to its slow growth and capnophilic nature using routine culture techniques [1,9,10,15,16,17,18,19]. *A. schaalii* has been identified in urine from patients with UTI or urosepsis, together with Gram-negative rods (*Citrobacter* sp., *Pseudomonas aeruginosa*, *Escherichia coli*, *Klebsiella* sp., *Proteus* sp.), Gram-positive cocci (*Aerococcus* sp., streptococci, and enterococci), or unspecified urinary tract microflora [9,12,14,18]. However, these observations are limited, because culture was used for detection, and the aims of case reports/case series were not to comprehensively identify *A. schaalii’s* concomitants.

Concurrently, it has been identified in patients with prostatism, prostate or bladder cancer [11,12,20,21,22,23], chronic inflammation [24,25], or urinary incontinence [6,26]. Thus, a connection to these conditions has been proposed [24,25,27,28]. However, all these conditions, symptoms, and diagnoses are supposed to be linked—either as the cause or consequence—to urobiome dysmicrobia [25]; therefore, the explanation for mutual bacterial relations including *Actinotignum* genus is getting more attention.

Urinary and ureteral catheter insertion is one of the most common urological interventions. At the same time, an inserted catheter is a condition changing the urinary tract micro-environment [29,30,31], promoting bacterial [30] and fungal colonization [32], and is a crucial risk factor for urinary tract infection (UTI).

*A. schaalii* was one of the most prevalent bacteria in a large cohort of urinary tract catheter samples, in earlier published observational study [30]. Kotaskova et al. [30], and others [10,16,19,33] were able to identify *A. schaalii* exclusively by broad-range molecular techniques. Concurrently, catheter presence was defined as a risk factor for *A. schaalii* caused UTI [6,9,19,34], possibly leading to bacteriemia [9,35] or even urosepsis [6,9,11,12]. Pedersen et al. [35] reported that 24.6%, and Sandlund et al. [9] that even 76.5% of patients with *A. schaalii* bacteriemia were catheterized. At the same time, urine collected via catheter is a material commonly analyzed for bacteria presence [15,17,19]. 

Few reports alerting this species’ potential importance in urinary tract polymicrobial communities and its role in catheter biofilms have appeared to date [27,36,37]. None of them were focused on urinary and ureteral (double-J) catheters, although *A. schaalii* had already been reported in both these materials [12,18,22,37]. Thus, we present observational study focused on *A. schaalii* on urinary (UC) and ureteral (DJC) catheters. Our aims were to: (i) characterize *A. schaalii’s* prevalence in ureteral/urinary catheter biofilms and urine samples; (ii) characterize the *A. schaalii’s* association with other bacteria in biofilm communities, thus suggest its concomitants; (iii) characterize *A. schaalii’s* association in ureteral/urinary catheters and urine with patients’ conditions and samples’ characteristics. In this study, we combined several methodological detection approaches. While culture- and PCR-based techniques were used in a wider set of samples providing qualitative information (presence/absence) at both patient and sample level, next-generation sequencing (NGS) revealed quantitative information (abundance) and thus enabled a deeper analysis in a subset of samples.

## 2. Materials and Methods

### 2.1. Study Design and Data Collection

The cohort of 133 patients and 347 proximal and distal catheter tip sonicate (in case of DJC), catheter sonicate (in case of UC), and corresponding urine samples from our previous study [30] were inspected. Repeated sampling and patients with infective diagnosis were excluded and the remaining 297 specimens from 128 patients were subjected to further analyses. Data about age, sex, type of catheter (DJC vs. UC), proximal or distal tip (in case of DJC), source material (sonication fluid vs. urine), and patients’ diagnoses were collected (for details, see Table 1 and Table S1). The study was approved by St. Anne’s University Hospital’s Ethics Committee (30.6.2015). No informed consent was required because neither human cells nor human tissues were processed and no procedure in addition to standard care was performed.

The pre-analytical catheter sonication was used to release microbes from biofilms. Sonication fluids and urines were inoculated onto the media set and cultured routinely. Moreover, sonication fluids were used for DNA extraction. The detailed laboratory procedure is described in our previous study [30]. All 297 samples were analyzed using culture techniques and Polymerase Chain Reaction followed by Denaturing Gradient Gel Electrophoresis and Sequencing (PCR-DGGE-S).

Twenty-nine samples were analyzed using next-generation amplicon sequencing, targeting the V3-V4 of *16S rRNA* [38]. In our previous study, *16S rRNA* amplicon sequencing was used as a reference method to evaluate methodological approaches, but here we interpret molecular techniques’ results including abundance information, thereby we provide new perspectives to the previous interpretation [30]. Moreover, advanced statistical techniques were applied to analyze culture and PCR-DGGE-S results, as well. Significance level α was set at 0.05 for all tests unless stated otherwise.

### 2.2. Preanalytical Procedure

The urine was obtained via catheter before displacement. After DJC or UC aseptical removal, the 5 cm long tips (proximal and distal part of DJC and the UC distal part) were snipped off and placed into 5 mL of Brain Heart Infusion (BHI) and Wilkins-Chalgren broth (Oxoid, Hampshire, UK) and sonicated (2 × 5 min interspaced by 2 min of vortexing). Sonication fluids and urine samples were used for inoculation and bacterial DNA extraction.

### 2.3. PCR-DGGE-S and Culture

#### 2.3.1. PCR-DGGE-S

The V3-V4 variable *16S rRNA* (~460 bp) regions were amplified using eubacterial primers FP338GC [39] and RP772 [40], forward primer was extended by GC-clamp at the 5′ end. The total volume of 30 µL of HotStarTaq Mastermix (Qiagen, Hilden, Germany), MgCl_2_ (1.5 mM final concentration), primers (0.5 µM final concentration of each primer), 8-methoxypsoralen (0.16 mM, 8-MOP, Sigma-Aldrich, St. Louis, MO, USA) were prepared. Mixtures were incubated at 4 °C for 1.5 h and exposed to UVA (365 nm) for 7 min (30 J/cm^2^) in UV-crosslinker for decontamination by the 8-MOP. After decontamination, 5 µL of DNA was added and PCR was performed. Initial denaturation at 95 °C last 15 min; 35 cycles of denaturation at 94 °C for 30 s, primer annealing at 59 °C for 1 min, extension at 72 °C for 1 min were followed by prolonged final extension at 72 °C for 30 min to avoid artificial PCR products formation.

PCR products were separated by DGGE (INGENYphorU-2x2 aparatus, Ingeny, Amsterdam, The Netherlands). A 6% polyacrylamide (37:1 AA:BAA, Sigma-Aldrich, St. Louis, MO, USA) with the 30–60% denaturing gradient (7 M urea and 40% formamide in 100% solution (Sigma-Aldrich, St. Louis, MO, USA)) was used in a 0.5xTAE running buffer. Gels were electrophoresed at 60 °C at a voltage of 12 V for 30 min, subsequently at 120 V for 15.5 h. Finally, the gel was stained with ethidium bromide for 20 min and documented. Visible bands were eluted overnight in 50 µL of sterile water, after excision. Re-amplification was performed using forward primer with no GC clamp. Products were visualized on 2% agarose gel, extracted from the gel by QIAquick Gel Extraction Kit (Qiagen, Hilden, Germany) and sequenced with ABI PRISM 3130 Avant Genetic Analyzer (Life technologies, Carlsbad, CA, USA).

#### 2.3.2. Culture

All sonication fluids and urine samples (1–100) were inoculated into a set of solid media: Blood Agar with 7% sheep’s blood (Oxoid, UK), Endo Agar (Imuna Pharm, Šarišské Michaľany, Slovakia), Wilkins Chalgren Anaerobic Agar with 7% sheep’s blood and vitamin K (WCHA, Oxoid, Hampshire, UK). UCs, DJCs and urine samples were inoculated in addition into Blood Agar with 10% of NaCl, Blood Agar with Amikacine (32 mg/L) (Oxoid, Hampshire, UK), and UriSelect 4 (Bio-Rad, Hercules, CA, USA). Plates were assessed for microbial growth after cultivating at 37 °C for 48 h. The WCHA was cultivated in an anaerobic atmosphere (80% N_2_, 10% CO_2_, and 10% H_2_; Anaerobic Work Station Concept 400, Ruskinn Technology, Bridgend, UK) at 37 °C for 7 days. All isolated strains were identified biochemically or using MALDI-TOF MS (Biotyper with FlexControl 3.4 software, Bruker Daltonics, Billerica, MA, USA), according to the manufacturer’s instructions. For details see [30].

#### 2.3.3. Culture and PCR-DGGE-S Data Analysis and Interpretation

Culture and PCR-DGGE-S results from all 297 specimens of 128 patients were combined, and the species matrix was constructed as PCR-DGGE-S and culture results disjunction. For this purpose, culture results in the form of presence/absence data were used (see Supplementary Tables S2 and S3).

Patients were considered *A. schaalii*-positive (*As+*) if the bacterium was detected at least in one sample; otherwise, they were considered *A. schaalii*-negative (*As−*). Statistical assessment was carried out in the R environment [41]. Fisher’s exact test was used to test the association between categorical variables with small numbers such as sex, type of catheter, presence/absence of a diagnosis. Moreover, Fisher’s exact test was applied to define species/groups of species more often presented in *As+* than *As−* patients, following the principle of indicator species analysis [42]. A non-parametric Wilcoxon rank-sum test or Kruskal–Wallis test was used to test two or more selections of continuous variable (age), respectively.

Permutational Multivariate Analysis of Variance Using Distance Matrices (PERMANOVA) with Bray–Curtis distance was performed to test the difference in species consortia composition between *As*+ and *As−* patients and Non-Metric Multidimensional Scaling (NMDS) was used to plot patients according to their species composition by approximating rank distances in a two-dimensional space. In these analyses, *A. schaalii* was excluded from the species matrix and treated as an explanatory variable.

Another NMDS diagram was created to display the relationships between species, including *A. schaalii*. The same procedure was used except that the variation in species composition was reduced by keeping only high-diversity patients (those with at least four species), and non-rare species (those found in at least five patients); in this analysis, *A. schaalii* was not treated as an explanatory variable. This led to extreme cases represented by species poor patients and rare species being eliminated. Using the same filtering, NMDS plots showing patients were created and all non-rare species presence/absence information was projected into these plots.

### 2.4. 16S rRNA Amplicon Sequencing

The V3-V4 region of *16S rRNA* (~460 bp long) in 29 samples was amplified using the previously published degenerated primers [38] with inner tags to distinguish the particular samples. Following the Illumina MiSeq standard protocol, PCR products were determined on 1.5% agarose gel and Agencourt AMPure XP beads (Beckman Coulter Genomics, Brea, CA, USA) were used to clean the PCR products according to the manufacturer’s recommendations. Results from the Qubit dsDNA HS Assay Kit (Invitrogen, Waltham, MA, USA) microplate reader Synergy Mx (BioTek, Winooski, VT, USA) were used to assess the cleaned PCR products’ concentration to pool them equimolarly (those with different inner tags). Pools were indexed with Nextera XT Library Preparation Kit (Illumina, San Diego, CA, USA), purified with Agencourt AMPure XP beads and finally pooled. The prepared library’s integrity was analyzed by a 2100 Bioanalyzer Instrument (Agilent Technologies, Santa Clara, CA, USA), and the concentration was measured with qPCR before sequencing (KAPA Library Quantification Kit, Roche, Switzerland). Sequencing was performed with the Miseq reagent kit V3 (2 × 300 bp, pair-end sequencing) using a MiSeq 2000 instrument according to the manufacturer’s instructions (Illumina, San Diego, CA, USA).

The whole procedure, including bioinformatics analysis empowering the QIIME is described in our previous study [30]. Briefly, pair-end reads passing quality control were merged using the fastq-join method in QIIME 1.9.1 [43]. Data were demultiplexed, barcodes and primers were trimmed in R. OTUs (Operational Taxonomic Units) were constructed as clusters of >97% sequence similarity using QIIME. Chimeras were detected with UCHIME in USEARCH v6.1.544 [44] and excluded. Taxonomy was assigned to each OTU based on the SILVA 123 reference database [45].

#### *16S rRNA* Amplicon Sequencing Data Analysis and Interpretation

To provide results: (i) comparable to PCR-DGGE-S and culture; (ii) interpretable in a clinical context, we aimed for species, genus and higher taxonomic levels in further analyses.

Besides Age, Sex, Type of catheter (DJC vs. UC), and Material (urine vs. sonication fluid), data about 3 diagnoses: hydronephrosis, malignant prostate neoplasm (prostate cancer), and any malignancy, entered further analyses because of a sufficient number of cases (metadata see in Supplementary Table S4).

QIIME results were visualized, analyzed, and statistically tested in Calypso v 8.84 [46], empowering the R environment. Besides the original matrix with *A. schaalii* abundance information (see Supplementary Table S5), an extra matrix excluding *A. schaalii* read counts were prepared to estimate residual communities when appropriate (residual matrix see in Supplementary Table S6). On the OTU, species, and genus taxonomic level, all rows containing “*Actinotignum*” or “*Actinobaculum*” were extracted from the matrix. It is important to note, that *A. schaalii* was the only identified species from *Actinotignum* genus in our dataset. *Actinotignum* genus read counts were subtracted from the appropriate higher taxonomic ranks’ taxa. An explanatory variable about *A. schaalii* presence/absence was added to the metadata.

Clustered stack bar charts for initial visualization and inspection were constructed using total sum scaling normalization. For further analyses, taxa with less than 0.01 percent relative abundance across all samples were filtered out and the centred log-ratio transformation was applied, if not mentioned differently.

For exploratory analysis and to identify associations between community composition and environmental variables, Principal Component Analysis (PCA) was empowered and clustered heatmaps were constructed. Moreover, the multiple regression model was used to inspect the association between each detected genus with each of the explanatory variables (including *A. schaalii* presence/absence) in the original matrix. To explore differently abundant taxa across *As+* and *As−* samples from the transformed residual matrix were defined by ANOVA, and a comparison of the two selections were made by Wilcoxon rank-sum test.

An original matrix with non-transformed, non-filtered and rarefied read counts was used to define richness-based α-diversity indices. Richness, ACE, and Chao1 index were used to evaluate diversity at species and genus levels. Shannon’s and Simpson’s index, and Shannon’s evenness (Evenness) were estimated at the genus and the species level, employing both matrices (original and residual). Between-group α-diversity indices’ variations in *As+* and *As−* samples were tested using non-parametric Wilcoxon rank-sum test. To identify associations between microbial original communities’ diversity indices (species-based Richness, Shannon’s index, Simpson´s index, and Evenness) and all available explanatory variables, the multiple regression was applied.

To explore the β-diversity, non-parametric Analysis of Similarities (ANOSIM) using Bray–Curtis distance metric describing community dissimilarities, and Bray–Curtis permutational manova (PERMANOVA) were used to reveal the statistical significance and to test whether the variance in community composition can be attributed to *A. schaalii* presence/absence or/and other explanatory variables. In order to summarize the linear relationship between components the residual matrix and a set of explanatory variables, (including *A. schaalii* presence/absence), a supervised multivariate transformation-based Redundancy analysis (tb-RDA) was performed and explained variance values for multiple variables were assessed.

## 3. Results

### 3.1. Culture and PCR-DGGE-S

#### 3.1.1. *A. schaalii* and Explanatory Variables

**Figure 1:**
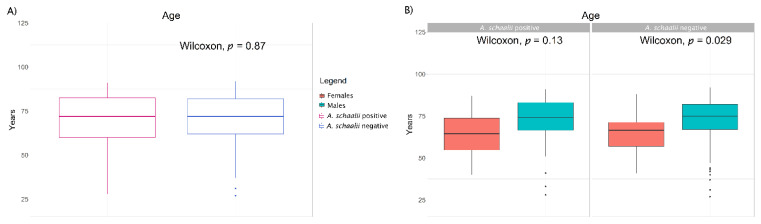
Age of *A. schaalii*-positive and -negative patients. Figure 1 shows the difference between *A. schaalii*-positive and -negative patients (**A**), and males and females (**B**). Legend between pictures is common for both. Wilcoxon rank-sum test was used to reveal differences between two selections (*p*-values are in plots). (**A**) No difference between the age of *A. schaalii*-positive and -negative patients was revealed. (**B**) No difference in *A. schaalii*-positive males’ and females’ age was observed (left side, *p* = 0.13), but, *A. schaalii*-negative males were significantly older than females (right side, *p* = 0.029).

In total, 1078 representatives of 143 species and 59 genera were identified. We observed no significant difference comparing *A. schaalii* prevalence in catheters and corresponding urine samples, in both UCs and DJCs (Table 1). In total, 32.3% of males and 37.5% of females were *A. schaalii* positive, no significant difference was observed, even regarding the type of material (see summary below Supplementary Table S1). We observed a higher age in *A. schaalii*-negative males than females (*p* = 0.029), see Figure 1B.

Considering the whole patient dataset, we observed a similar *A. schaalii* prevalence in both subgroups of DJC (30.9%) and UC (35.6%) patients (see summary below Supplementary Table S1). Therefore, we assume *A. schaalii* is not inclined to any type of catheter (UC or DJC), which is also the case of other bacteria, such as *Escherichia* sp. (present in 48.0% UC and 32.7% DJC patients, *p =* 0.1036), while the other most prevalent representants in our dataset showed a significantly higher prevalence in UC than DJC patients, such as *Enterococcus* sp. (detected in 80.8% UC and 41.8% DJC patients, *p* < 0.0001) or *Proteus* sp. (present in 49.3% UC and 16.4% DJC patients, *p* < 0.0002), see Supplementary Table S3 for details.

When addressing patients’ diagnoses, we observed a statistically higher *A. schaalii* prevalence in patients with ureter stricture associated hydronephrosis (18.6%, *p* < 0.0206, see Table 2) than in the remaining patients (4.7%). No other tested diagnosis (Table 2) was shown to be associated with different *A. schaalii* prevalence. Our results did not suggest *A. schaalii*’s prevalence in relation to malign prostatic neoplasia (Table 2).

#### 3.1.2. Diversity of Bacterial Communities

**Figure 2:**
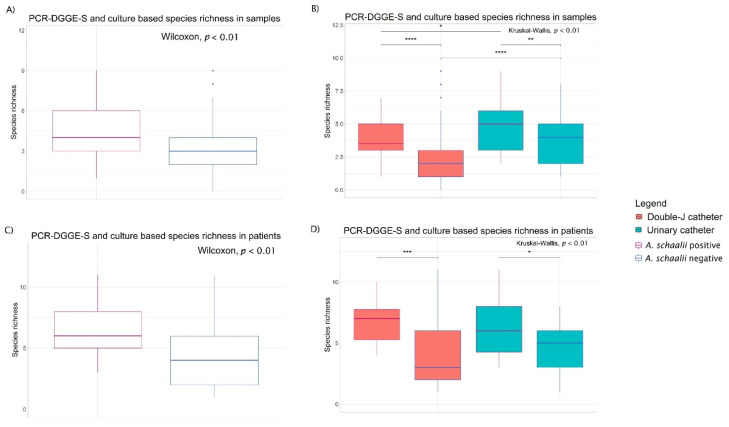
Species richness. Figure 2 shows the difference between species richness. The legend on the right side is common for all plots. Differences among more than two groups were tested by Kruskal–Wallis test (*p*-value is in the plot). Wilcoxon rank-sum test was used to reveal differences between two selections (in plots in case of significance). * 0.01 < *p* ≤ 0.05; ** 0.001 < *p* ≤ 0.01; *** 0.0001 < *p* ≤ 0.001; **** 0.00001 < *p* ≤ 0.0001. (**A**) Plots show the comparison of *A. schaalii*-positive/-negative samples, and positive/negative ureteral (DJC)/urinary (UC) (**B**). Significantly higher richness in *A. schaalii*-positive samples was observed; the higher richness was observed in *A. schaalii*-positive DJC and UC samples compared to those that were negative. Concurrently, more species were detected in UCs than DJCs. (**C**) Plots show the comparison of *A. schaalii*-positive/-negative patients and positive/negative patients with DJC/UC (**D**). The significantly higher richness in *A. schaalii*-positive patients was observed; the higher richness was observed in both *A. schaalii*-positive DJC and UC patients compared to those who were negative.

We observed significantly higher richness in *A. schaalii*-positive samples overall and both in DJC and UC samples separately, compared to those that were negative (for details see Figure 2A). Concurrently, more species were detected in UCs than DJCs, regardless of *A. schaalii* positivity (see Figure 2B). Similar trends were apparent in patients (for details see Figure 2C,D).

**Figure 3:**
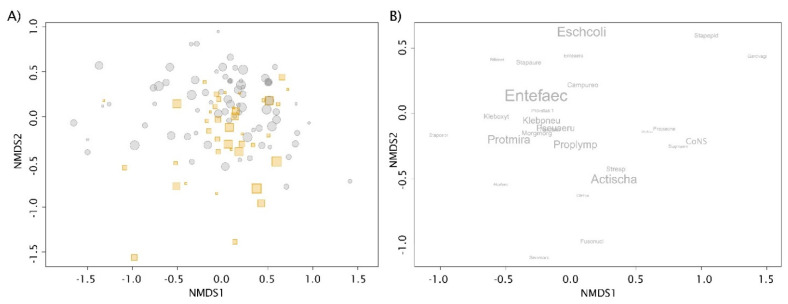
Non-Metric Multidimensional Scaling (NMDS) ordinations. (**A**) NMDS ordination of 128 patients according to their species composition (stress = 0.18). Positive (orange squares) and negative (grey circles) *A. schaalii* patients are distinguished. *A. schaalii* was excluded from the species data set and was treated as an explanatory variable. The symbol size is proportional to richness (number of bacteria detected in the patient). Patients from both groups were partly separated, but the groups overlapped. This indicates the species composition did not differ much between the groups. (**B**) Bacteria positioned in NMDS ordination of samples as weighted sample score averages (stress = 0.18). This NMDS diagram was created to display the relationships between species, including *A. schaalii*. Only high-diversity patients (those with at least four bacteria) and non-rare species (those with at least five occurrences) were kept to calculate the ordination. This led to the elimination of extreme cases represented by rare species and patients with a low number of detected representatives. The font size is proportional to the occurrence frequency in the studied population.

Estimating β-diversity, Non-metric Multidimensional Scaling (NMDS) ordination of 128 patients showed only partial *As*+ and *As*− patient separation. This indicates the species composition does not differ much between these groups (see Figure 3A). Regarding the *A. schaalii* presence as an explanatory variable, a statistical difference between *As*+ and *As*− patients was observed (PERMANOVA, *p =* 0.03, r^2^ = 0.01867) in the whole dataset of 128 patients. However, the presence of *A. schaalii* explained just 1.6% of the species composition matrix variability. Species positioned in NMDS ordination of all samples showed the importance of species and connections among them (see Figure 3B). Although the only statistically important co-occurrence with *A. schaalii* was observed in the case of closely positioned *P. lymphophilum* (*p* < 0.00001), the *A. schaalii* proximity to *Streptococcus* sp., *Pseudomonas aeruginosa,* and *Fusobacterium nucleatum* was obvious as well (for results in patients see Table 3). For the NMDS ordination of high-diversity patients with projected 27 non-rare species—see Supplementary Figure S1.

#### 3.1.3. Concomitant Species of *A. schaalii*-Positive Patients

Further, we defined a group of species more often present in *As*+ patients: *Propionimicrobium lymphophilum*, *Fusobacterium nucleatum*, *Alcaligenes faecalis,* and *Streptococcus* spp. Any representative of this concomitant species group was detected in 67.4% of *As*+ patients, contrary to 23.5% of *As*− patients (*p* < 0.0001), for details, see Table 3. No statistical difference was shown testing the effect of other possible explanatory variables such as sex, age, or diagnosis on the prevalence of the abovementioned concomitant species.

### 3.2. 16S rRNA Amplicon Sequencing

#### 3.2.1. Exploratory Analyses

**Figure 4:**
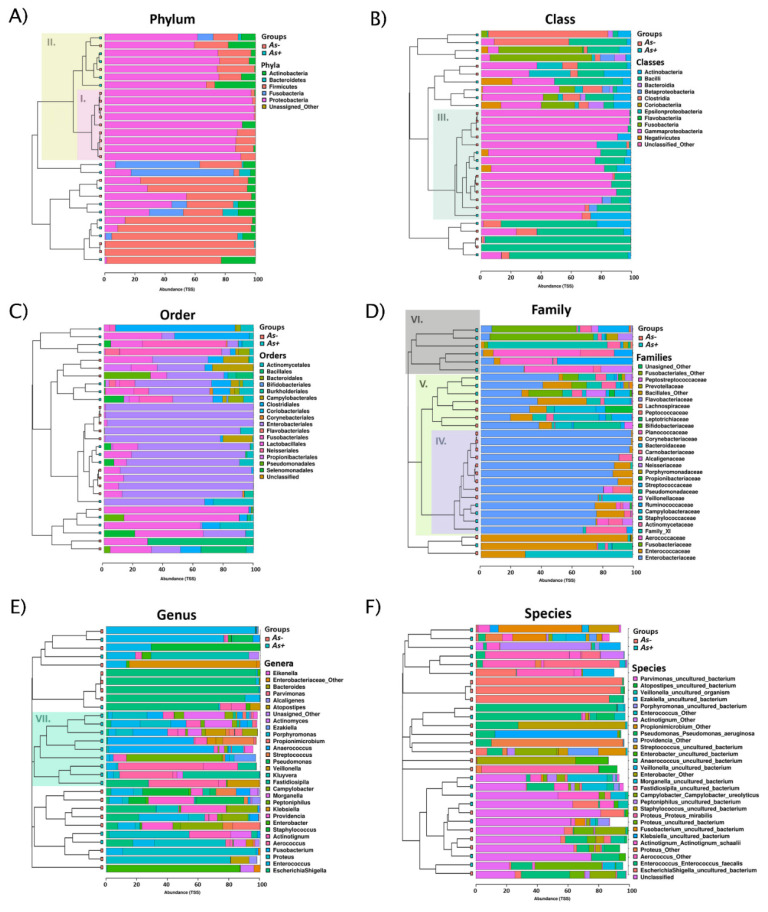
Stacked clustered bar charts.Bar charts showed the relative abundance (x-axis, total sum scaling normalization) of detected taxa (see the legend on the right side of each bar chart) at different taxonomic levels (**A**–**F**). Samples (rows) were hierarchically clustered and *As+* and *As*− samples were labelled (green and pink). For family (**D**), genus (**E**), and species (**F**) level, only the 30 most abundant taxa were depicted. Sample clusters were marked by roman numerals (I.–VII.). Noteworthy, clustering *As*− samples was more apparent at higher taxonomic ranks (**A**,**B**,**D**), while *As+* samples made clusters at lower taxonomic ranks (**D**,**E**). Nine (60% of *As−* samples, 100% of cluster) and 11 (73.3% of *As*− samples, 68.8% of cluster) *As*− samples were clustered (I., II.) at the phylum level (**A**), commonly with the high abundance of Proteobacteria. Moreover, 10 (66.7% of *As−* samples, 71.4% of cluster) *As*− samples remained clustered (III.) at the class level with Gammaproteobacteria as the most abundant class. Enterobacteriaceae was the most abundant family of cluster IV. consisting of 9 (60% of *As*− samples, 69.2% of cluster) *As*− samples. There were 13 (86.7% of *As−* samples, 65% of cluster) *As*− samples grouped in the V. cluster, characterized by Enterobacteriaceae family presence, but not necessarily dominant over others. At the family and genus level, the *As+* samples clustered together (see clusters VI, VII). These clusters were defined by high taxonomical diversity, absence of distinctly dominant taxa, and presence of rare genera (rest up to 100%).

Amplicon sequencing revealed the presence of 97 species from 58 genera in 29 samples (see Supplementary Table S5) with an average sequencing depth of 4515 reads per sample. For an overview of relative taxa abundance from phylum to species taxonomic level, see Figure 4. Clustered bar charts showed the clustering of *As+* samples at family, genus, and species level. At genus level, the created clusters were defined by higher taxonomical diversity, distinctly dominant taxa’s absence and rare genera’s presence. *As*− samples clustered at higher taxonomical levels and generated clusters were defined by Enterobacteriaceae’s dominance.

**Figure 5:**
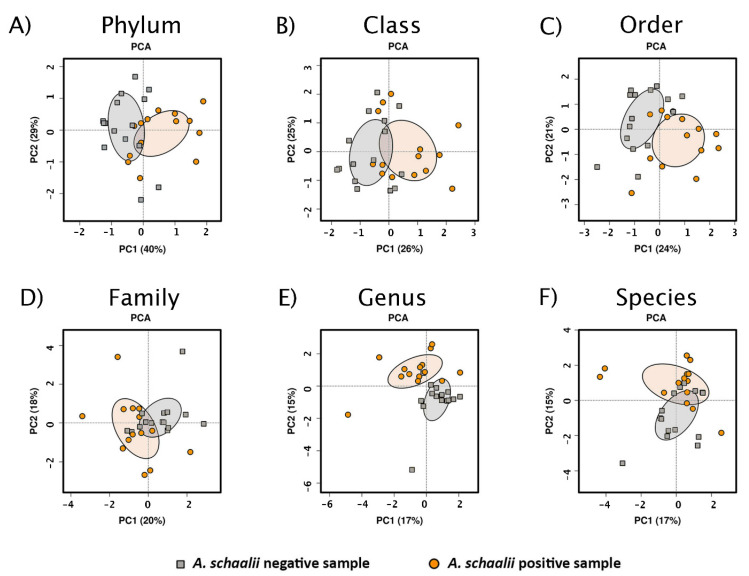
Principal Component Analysis (PCA) biplots with *A. schaalii* presence/absence projected. PCA biplots showed unsupervised ordinated sample clustering of different taxonomic ranks (**A**–**F**), *A. schaalii* presence/absence was projected and 95% confidence interval was depicted by filled ellipses. The clustering according to a projected variable was visible, most apparently at the genus level (**E**), although the explained variability increased with higher taxonomic ranks (compare explained variability from species (**E**) to phylum level (**A**)).

**Figure 6:**
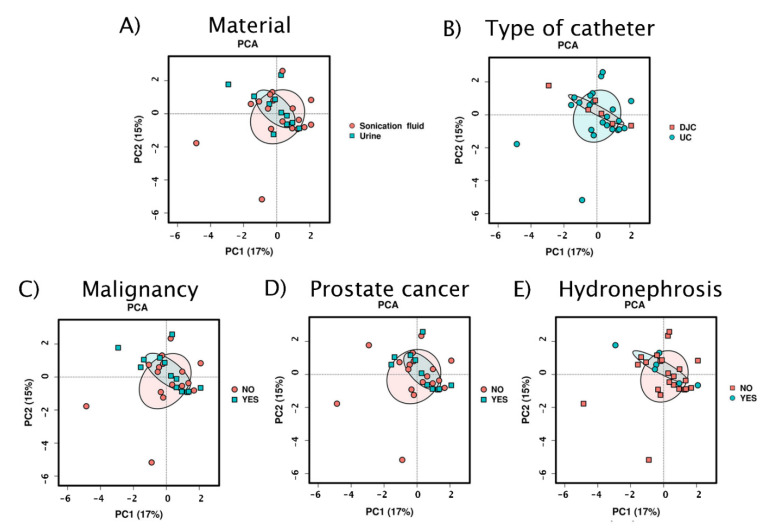
PCA biplots (genus level) with variables other than *A. schaalii* presence/absence projected. PCA biplots showed unsupervised ordinated sample clustering with projected explanatory variables (**A**–**E**). Filled ellipses represent a 95% confidence interval. Clustering according to a projected variable was not apparent, therefore, the most explained variation in all variables could be attributed to *A. schaalii* presence/absence (compare to Figure 5).

PCA ordinated samples were visualized with biplots, those with the projected variable—*A. schaalii* presence/absence—are shown in Figure 5. Separating *As+* and *As*− samples alongside the PC1 and/or PC2 axis is obvious at all taxonomic levels, but mostly at the genus level. At the same time, none of the other known variables could explain the variability in community structure better than *A. schaalii* presence/absence (for genus level see Figure 6). This indicates, no confounding factor affected the community composition in *As+* and *As*− samples, and the community composition was driven by *A. schaalii* presence/absence.

**Figure 7:**
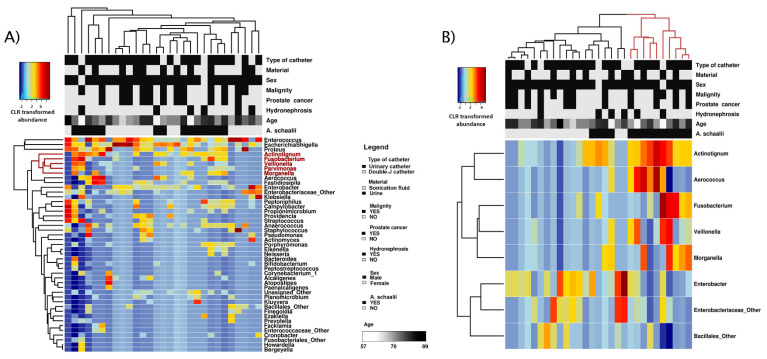
Heatmaps and cluster analysis. Heatmaps showed the CLR-transformed abundance of genera (rows). Unsupervised sample grouping (columns) with similar community composition and genera with similar abundance across samples into clusters was achieved by hierarchical clustering. Explanatory variables’ values presented as a separate heatmap on top of both heatmaps, the legend is between pictures and is common for both heatmaps. (**A**) Heatmap showed the abundance of all detected genera (*n* = 45). Those genera *Actinotignum* was clustered with are in red (*Fusobacterium*, *Veillonella*, *Parvimonas*, *Morganella*). Regarding samples, no cluster consisting of exclusively *As+/As−* samples was apparent. At the same time, the explanatory variables’ factors seemed to be spread equally among all clusters and *As+*/*As−* sample groups; *A. schaalii* presence did not seem to explain the clustering better than any other explanatory variables. (**B**) Only genera with significantly different abundance between *As+* and *As−* samples were shown (*p* = 0.05, ANOVA)—note the absence of *Parvimonas*. *Aerococcus*, *Fusobacterium*, *Veillonella*, and *Morganella* were genera co-occurring with *Actinotignum* across samples; *Enterobacter* and unspecified representatives from Enterobacteriaceae and Bacillales were more often present in *As*− samples, and less often present in *As+*. Ten *As+* samples (71%) were clustered in one cluster (red), and this cluster consisted of *As+* samples, exclusively.

**Figure 8:**
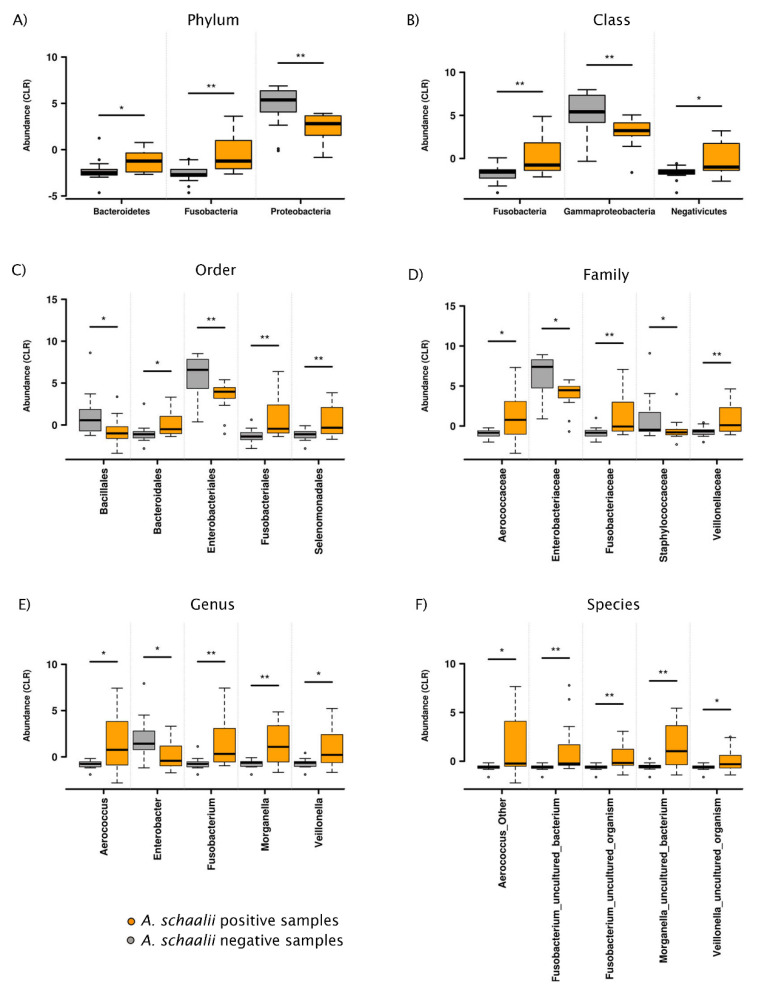
Taxa differently abundant across *As+* and *As−* samples. * 0.01 < *p* ≤ 0.05; ** 0.001 < *p* ≤ 0.01; CLR-transformed abundance boxplots are shown, only significantly different taxa were plotted. It was apparent that *A. schaalii* was mutually exclusive to *Enterobacter*, Enterobacteriaceae, Enterobacteriales, Gammaproteobacteria, Proteobacteria, and Staphylococcaceae family bacteria, and Bacillales order. On the other side, *Aerococcus*, *Fusobacterium*, *Morganella*, and *Veillonella* were genera with significantly higher abundance in *A. schaalii*-positive samples.

Heatmaps in Figure 7 showed detected genera and explanatory variable abundances. Clustering showed genera similarly abundant to *A. schaalii* (red cluster, Figure 7A) across samples: *Fusobacterium*, *Veillonella*, *Parvimonas*, *Morganella*. All of these genera, except for *Parvimonas*, had a significantly different abundance between *As+* and *As*− samples (see Figure 7B). CLR transformed taxa abundance differently abundant between sample groups (*As+* vs. *As−*) is in Figure 8.

The multiple regression model was used to inspect each detected genus’ association with each explanatory variable. Focusing on the *A. schaalii* presence/absence variable, seven genera were significantly associated with *A. schaalii* presence (*p* < 0.05), but no genus remained significantly associated after FDR correction for multiple testing. Regarding other variables, the only significant associations after FDR correction were identified in age and abundance of unspecified Fusobacteriales bacterium. A detailed list of *p*-values and *p*-values histograms are in Supplementary Table S7. Focusing on *A. schaalii* exclusively, no significant association between CLR transformed abundance and any other variable was observed (details and *p*-values are in Supplementary Table S7, for *A. schaalii* abundance plots and explanatory variables, see Supplementary Figure S2).

#### 3.2.2. Alpha Diversity

The overall higher α-diversity in *As+* samples, demonstrated by culture and PCR-DGGE (see above), was confirmed by Illumina sequencing (for details see Supplementary Figure S3). The observed richness was significantly higher in *As+* than *As−* samples both at the genus (*p* = 0.013) and species level (*p* = 0.012). Therefore, *A. schaalii* is a part of more diverse communities. None of the other richness-based indices (ACE, Chao1) were significantly higher at genus or species level, referring to a difference in α-diversity, but not in singletons, doubletons or rare taxa. All genus- and species-based metrics calculated from both matrices (original as well residual) were significantly higher in *As+* samples, except Simpson´s index (giving more weight to the dominant species, the presence of rare species causes small changes) derived from the residual matrix. This indicates *A. schaalii*’s abundance contributed to a higher Simpson´s index in the original matrix. To conclude, *A. schaalii* can be suspected as one of the dominant species in highly diverse *As+* samples.

The multivariable linear regression model revealed *A. schaalii*’s presence/absence to be the only factor significantly associated to α-diversity indices difference (genus-based Richness, Shannon’s index, Simpson’s index, Evenness) (for details see Supplementary Figures S4–S7). Therefore, we do not expect any of the tested variables to be confounding or affect α-diversity assessment.

#### 3.2.3. Beta Diversity

**Figure 9:**
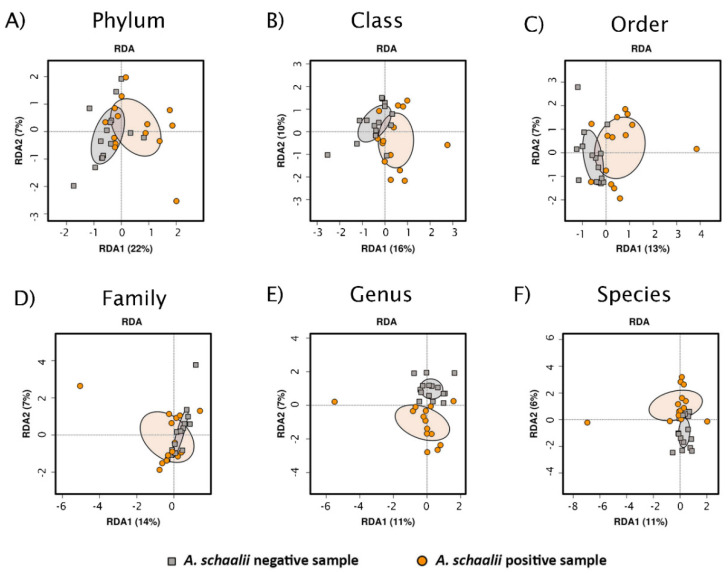
Redundancy Analysis (RDA) plots. Figure shows RDA plots at different taxonomic levels (**A**–**F**). The first two axes explaining the most variability are shown, the explained variability is in brackets. Ellipses are based on a 95% confidence interval and are shown for *As+* and *As−* samples.

Non-parametric Analysis of similarities (Anosim) showed the statistical difference in Bray–Curtis metric between *As+* and *As−* samples. Moreover, the intra-group community structure variation in each group (*As+* and *As−* samples) was lower than between-group variation (*p* = 0.001), for details see Supplementary Figure S8. PERMANOVA revealed a significant difference between *As+* and *As−* samples at species, genus and family level (see Supplementary Table S8). The variation in the community composition can be attributed to *A. schaalii* presence/absence at each of tested taxonomic levels; age and sex seem to contribute to the residual community composition too. A similar conclusion can be made from tb-RDA results: *A. schaalii* presence/absence and age were the only two variables significantly associated with variation in the residual data matrix, although the unexplained variance is relatively high (RDA biplots with *A. schaalii* presence/absence projected are in Figure 9, details to explained variability and *p*-values are in Supplementary Table S9).

## 4. Discussion

*A. schaalii* prevalence does not differ significantly between males and females [4], as we report in this study. However, a slightly higher prevalence was observed in females (37.5%) than males (32.2%) (see Table 1). The male/female ratio of positive patients only with an *A. schaalii* infection is usually reported 1 to 1.5 [4], up to 4.7 [9]. We report the ratio of 2.6 from *A. schaalii* colonized individuals with no infection.

Increased *A. schaalii* prevalence in UTI patients is commonly associated with advanced age [4,36]. We suppose that *A. schaalii*‘s position is transformed from a bystanding concomitant to a UTI agent or co-agent in the dysmicrobic urinary tract environment and/or in the elderly population with immune senescence. Age-associated alterations in innate immunity could facilitate an otherwise harmless, host-adapted *A. schaalii* strain to establish infection or co-infection by actively suppressing the local immune responses in the urinary tract [47,48]. Thus, a lack of infectious patients in our dataset might be the reason why we did not observe *A. schaalii* positivity increasing with age.

Further, we observed no significant difference in *A. schaalii* prevalence between DJC and UC. Since the species richness was higher in UC with both approaches (presence/absence-based PCR-DGGE-S combined with culture, as well as abundance-based NGS), as expected (see Figure 2, Supplementary Figures S4–S7), we do not assume any underestimation in UCs’ species richness. This finding can refer to *A. schaalii*’s ability to be present in any catheters’ biofilm (including nephrostomy) [2,9,12,18,22,37], regardless of the catheter type. To confirm this hypothesis in non-infected but colonized patients, the cohort with non-catheterized individuals should be examined, although the case series and clinical observations have already defined unspecified catheterization as a risk factor for *A. schaalii* infection [6,9,12,19,34,35].

The urobiome α-diversity was found higher in older individuals and in those with fewer UTI episodes in their history [49], referring to its supposedly protective function against UTI [49,50]. Interestingly, Buhmann et al. [37] characterized one of 11 catheter encrustation urotypes by *Actinotignum* presence. This urotype was defined by the 3rd highest mean Shannon’s index of all 11 urotypes. In concordance with these findings, we identified a significantly higher species richness in *A. schaalii*-positive samples by both methodical approaches (see Figure 2 and Figure 4 and Supplementary Figure S3). NGS confirmed higher Evenness, Shannon’s, and Simpson’s indices in *As+* samples, and the trend of α-diversity and age association with *A. schaalii* presence, expressed mainly in Richness and Simpson’s index (see Supplementary Figures S4–S7), was observed too.

Various microbial communities and urotypes are reported to be associated with non-infectious disorders and conditions [25], such as bladder cancer [27,51], prostatic neoplasia [24], or urinary incontinence [24,27]. We report significantly different β-diversity in *A. schaalii*-positive and -negative samples (defined by NGS, see Supplementary Table S8) and patients (defined by PCR-DGGE-S and culture, see Chapter 3.1.3). In addition, Anosim via Bray–Curtis index and RDA analysis (Figure 8) confirmed differences between sample communities. Besides *A. schaalii* presence/absence, the Age (all levels except phylum) and Sex (at family level) drove the community composition, although less strongly (compare R^2^ in Supplementary Table S9). The catheterization or nephrostomy, hyperplasia or malignancy, and stricture are the commonly reported urinary tract associated comorbidities for patients suffering from an *A. schaalii* infection [9,22]. Our results did not prove the *A. schaalii* prevalence was related to bladder or prostate cancer [1,12,22,24,27], possibly due to the limited patient group sizes (Table 1). The only significantly associated condition observed in our study was the hydronephrosis with ureter stricture, described earlier as a comorbidity [9,14,22,23,35].

The significant difference between *As+* and *As*− samples’ β-diversity indicates that there are bacteria mutually exclusive/co-occurring to *A. schaalii*, as proved by many reports of poly-bacterial infections [5,6,7,8,9,10]. We identified *P. lymphophilum* and *Fusobacterium nucleatum* both separately and combined with *Alcaligenes faecalis* and *Streptococcus* spp. as concomitants to *A. schaalii* in patients, based on the presence/absence data (see Table 3, Supplementary Figure S1). At the same time, we showed *Fusobacterium*, *Veillonella*, *Morganella*, and *Aerococcus* are co-occurring and *Enterobacter* and bacteria from Staphylococcaceae are mutually exclusive to *A. schaalii.*

Except for opportunistic *Alcaligenes faecalis*, each of the above-mentioned species was already detected together with *A. schaalii* in patients with different conditions. Focusing on infections, *P. lymphophilum*—a rare UTI agent [52,53]—was co-detected with *A. schaalii* in a bacteraemia patient with urinating difficulties [5]. On the other hand, *Aerococcus* is a common uropathogen [54], which was identified together with *A. schaalii* in urosepsis [6]. Pedersen et al. [35] recognized *Aerococcus* sp. as the most common *A. schaalii* concomitant, found in 9 out of 29 polybacterial blood samples of septic patients and Bank et al. [14] detected it in 2 out of 10 *A. schaalii*-positive patients. Tschudin-Sutter et al. [12] noted *Veillonella* and *A. schaalii* in two blood samples from septic patients with intra-abdominal infections; *Morganella* was co-detected in ankle osteitis [13], and streptococcus in subcutaneous cyst [13] and blood [9]. In concordance with our results, and regarding more complex infections, Moustafa et al. [55] reported *A. schaalii* as a dominant species in UTI urine samples together with *Veillonella*, *Streptococcus*, *Aerococcus* and others. Moreover, a cluster of samples dominated by these agents was defined by higher α-diversity (genera richness) than the cluster defined by *Enterobacter* genus dominance [55]. This finding corresponds to decreased α-diversity in *A. schaalii*-negative samples and mutually exclusive relationship to *Enterobacter* genus, observed in our study.

In the DJC biofilm of the asymptomatic patient, Yu et al. [56] observed *Actinotignum massiliense* (closely related to *A. schaalii*), together with *P. lymphophilum*, *Streptococcus agalactiae, Aerococcus urinae* and three others. Inflammation inducing bacteria, including *A. schaalii*, *P. lymphophilum*, *Streptococcus anginosus*, and 3 other species, have been suggested as being associated with prostate cancer [24]. In another study, *A. schaalii* with protumorigenic *Fusobacterium* sp., and 6 other genera were detected more often in the urine of bladder cancer patients. Moreover, *P. lymphophilum* has been identified as a source of androgens in the body, potentially contributing to prostate cell proliferation and therefore prostate cancer. 

Defined in oral biofilms, Gram-positive cocci, such as various *Streptococcus* species, are typically reported as primary pioneering colonizers [57,58,59]. *Morganella moraganii* was also reported as a strong biofilm former [60] with appropriate genetic fimbrial and adhesion protein equipment [61]. *A. schaalii* related species from the same family—*Actinomyces naeslundii* [59] and *Actinomyces oris* [62] were recognized as initial or early phase colonizers. Aerococci—another Gram-positive cocci of *A. schaalii* concomitants—have recently been shown to form biofilms [63,64], however, the particular involvement in multispecies biofilm formation has not yet been clarified.

*F. nucleatum*, known for its elongated shape, serves as a mutualistic bridge organism, connecting primary colonizers to the typically anaerobic secondary colonizers [57]. *F. nucleatum* RadD adhesin and *Actinomyces naeslundii* interaction was proved [60]. Different from RadD, but unclearly defined other structures mediate *F. nucleatum* co-aggregation with early to middle stage colonizing *Veillonella*—another member of the *A. schaalii* related consortium [59,62,65]. Once adhered to the developing biofilm, *F. nucleatum* co-aggregates with secondary colonizers.

Bacterial cells within the biofilm engage not only in mechanistic, but also metabolic interactions, manifested as cross-feeding. *A. schaalii* is a lactic acid producer—similar to streptococci [66], or aerococci [64]—a complete glycolytic pathway is present leading to pyruvate production, which can be subsequently converted to lactate, acetate and ethanol [2]; a wide range of carbon sources, including pentose sugar (arabinose, ribose and xylose), hexose sugars (glucose), and disaccharides (maltose and sucrose) [2] can be utilised. Periasamy and Kolenbrander [65] suggest that *Veillonella*—recognized as an early colonizer—uses lactic acid from primary and other early colonizers for growth; thus, lactate produced by *A. schaalii* could be utilized in this way too. *F. nucleatum* is an asacharolytic organism, preferring amino acid fermentation as an energy source [57]; therefore, these species do not compete for resources. A possible explanation for chemical crosstalk between other defined bacterial concomitants is impeded by the absence of dedicated studies. For example, *Propionimicrobium lymphophilum’s* metabolic activity or genetic equipment has not been studied in depth. We hypothesize that *A. schaalii* acts as a colonizer of early biofilm formation stages, co-aggregating with *F. nucleatum*, and cohabitating with others.

Two clusters of tight adhesion genes (*tad*) in *A. schaalii*’s genome, encoding the machinery required to assemble pili, and genes for adhesive fimbriae construction [2] are present. Evidence showing, that *tad* loci are important for either early, middle or late colonization in various species, is accumulating [67]. However, we are aware that further experimental studies are needed for a deeper understanding of urinary tract biofilm communities, its concomitants and the interactions between them.

All these findings together with our results, support a presumption that *A. schaalii* and the described concomitants inhabit the urinary tract. Highly diverse communities may act protectively against UTI, probably by innate immune response activation, but comorbidities or risk factors can make *A. schaalii* and/or concomitants UTI causal agents. On a long-term scale, communities may modulate uroepithel by inflammasome, potentiating neoplasia progression. In fact, *A. schaalii* should be resistant to oxidative stress [2], which is an important factor in malignant cell transformation. The question remains whether *A. schaalii* survives due to its genetic equipment in the inflammatory microenvironment induced by co-habitants, or actively contributes to forming this microenvironment and therefore promotes cancerogenesis.

We are aware of a lack of studies dedicated to healthy urobiomes impeding the interpretation of patients’ urobiomes with any conditions. We believe a description and a better understanding of healthy and conditional/unhealthy urobiomes, including less known genera such as *Actinotignum*, could be utilized to alter urinary microbiota for better infectious as well as non-infectious condition management.

## 5. Conclusions

Our results did not show *A. schaalii* prevalence to be associated with sex, age, or type of catheter. However, in concordance to previous studies, significantly higher α-diversity in patients with *A. schaalii* was clearly shown, and a group of concomitant species—*Propionimicrobium lymphophilum*, *Fusobacterium nucleatum*, *Veillonella*, *Morganella*, and *Aerococcus*—was defined. On the other hand, *Enterobacter* and Staphylococcaceae were taxa identified as mutually exclusive to *A. schaalii*. Pending further exploration, our results showed higher *A. schaalii* prevalence in patients with ureter stricture associated hydronephrosis—previously described *A. schaalii* infection comorbidity. At the same time, we did not observe any connection to bladder or prostate cancer in our study. To conclude, we support the assumption of *A. schaalii*’s importance for catheterized patients and suggested *A. schaalii*’s inclination to a polymicrobial lifestyle with defined concomitants. To the best of our knowledge, we have provided the most comprehensive report dedicated to *A. schaalii* and its concomitants in the urinary tract.

## Figures and Tables

**Table 1:** Samples’ characteristics.

	Urinary Catheter-Related (UC)				Double-J Catheter-Related (DJC) ^1^				In Total (% of All Samples)
	*A. schaalii* Positive (% of Subtotal)	*A. schaalii* Negative (% of Subtotal)	Subtotal (% of All Samples)	*p*-Value ^2^	*A. schaalii* Positive (% of Subtotal)	*A. schaalii* Negative (% of Subtotal)	Subtotal (% of All Samples)	*p*-Value ^2^	
Urine samples	15 (23.9)	48 (76.1)	63 (46.7)	0.340	10 (18.9)	43 (81.1)	53 (49.1)	0.813	116 (47.8)
Catheter sonicates ^1^	23 (31.9)	49 (68.1)	72 (53.3)		12 (21.8)	43 (78.2)	55 (50.9)		127 (52.3)
All samples	38 (28.1)	97 (71.9)	135 (100)		22 (20.4)	86 (79.6)	108 (100)		243

^1^ Distal and proximal DJC tip were sonicated and analyzed separately (297 samples in total). However, merged *A. schaalii* results are shown in this table; ^2^ We tested by Fisher’s exact test whether there is a statistically significant association between *A. schaalii* presence and a sample type (urine sample vs. catheter sonicate), for UC and DJC samples separately.

**Table 2:** Patients’ characteristics.

	*A. schaalii* Positive ^1^, *n* = 43, (% of Positive Patients)	*A. schaalii* Negative, *n* = 85, (% of Positive Patients)	In Total (% of All Patients)	*p*-Value ^2^
Sex				
Males (median age ± SD)	31 (74.0 ± 15.4) (72.1)	65 (75.0 ± 15.3) (76.5)	96 (75 ± 15.3) (75.0)	0.667
Females (median age ± SD)	12 (64.5 ± 14.6) (27.9)	20 (66.5 ± 12.1) (23.5)	32 (66 ± 13.1) (25.0)	
**Type of Catheter in Patients**				
Double-J catheter	17 (39.5)	38 (44.7)	55 (43.0)	0.706
Urinary Catheter	26 (60.5)	47 (55.3)	73 (57.0)	
**Patients’ Diagnoses**				**Significance ^2^**
Renal colic	5 (11.6)	19 (22.4)	24 (18.8)	n.s.
Hydronephrosis	15 (34.9)	28 (32.9)	43 (33.6)	n.s.
Hydronephrosis with ureter stricture	8 (18.6)	4 (4.7)	12 (9.4)	*
Urolithiasis	13 (30.2)	31 (36.5)	44 (34.4)	n.s.
Prostatic or urinary tract cancer ^3^	10 (23.3)	23 (27.1)	33 (26.8)	n.s.
Bladder cancer	2 (4.7)	5 (5.9)	7 (5.5)	n.s.
Prostate cancer ^4^	7 (22.6)	13 (20)	20 (15.6)	n.s.
Other	2 (4.7)	5 (9.4)	7 (5.5)	n.s.
Other (none of mentioned above)	16 (37.2)	23 (27.1)	39 (30.5)	n.s.
**All Patients (% of 128 Patients)**	43 (33.6)	85 (66.4)	128 (100)	

^1^ Patient is considered as *A. schaalii* positive if at least one of his/her sample is *A. schaalii* positive; ^2^ We tested by Fisher’s exact test whether there is a statistically significant association between *A. schaalii* presence in patients (*A. schaalii*-positive vs. -negative patients) and sex (males vs. females), type of catheterization (urinary vs. ureteral), and respective diagnosis (present vs. absent). n.s.—not statistically important difference when comparing *A. schaalii*-positive and -negative patients. * 0.01 < *p* ≤ 0.05; ^3^ One *A. schaalii*-positive patient was diagnosed with prostate and kidney cancer; ^4^ 31 *A. schaalii*-positive and 65 *A. schaalii*-negative males were included; *p*-value and percent refer to male counts, exclusively.

**Table 3:** Concomitant species for *A. schaalii*-positive patients.

Concomitant Species ^1^	*A. schaalii* Positive, *n* = 43, (% of Positive Patients)	*A. schaalii* Negative, *n* = 85, (% of Negative Patients)	In Total (% of All Patients)	Statistical Significance ^2^
*P. lymphophilum*	19 (44.2)	10 (11.8)	29 (22.7)	***
*F. nucleatum*	7 (16.3)	3 (3.5)	10 (7.8)	*
*Streptococcus* spp.	9 (20.9)	10 (11.8)	19 (14.8)	n.s.
*Alcaligenes faecalis*	3 (6.9)	2 (2.4)	5 (3.9)	n.s.
*P. lymphophilum, F. nucleatum*	24 (55.8)	11 (12.9)	35 (27.3)	***
*P. lymphophilum, Streptococcus* spp.	26 (60.6)	18 (21.2)	44 (34.4)	***
*P. lymphophilum, A. faecalis*	21 (48.8)	12 (14.1)	33 (25.8)	***
*F. nucleatum, Streptococcus* spp.	12 (27.9)	11 (12.9)	23 (18.0)	n.s.
*F. nucleatum, A. faecalis*	10 (23.3)	5 (5.9)	15 (11.8)	**
*Streptococcus* spp., *A. faecalis*	12 (27.9)	12 (14.1)	24 (18.7)	n.s.
*P. lymphophilum, F. nucleatum, A. faecalis*	26 (60.5)	13 (15.3)	39 (30.5)	***
*P. lymphophilum, F. nucleatum, Streptococcus* spp.	27 (63.8)	18 (21.2)	45 (35.2)	***
*P. lymphophilum, A. faecalis, Streptococcus* spp.	28 (65.1)	20 (23.5)	48 (37.5)	***
*F. nucleatum, A. faecalis, Streptococcus* spp.	15 (34.9)	13 (15.3)	28 (21.9)	*
Any of indicator species	29 (67.4)	20 (23.5)	49 (38.3)	***
None of indicator species	14 (32.6)	65 (76.5)	79 (61.7)	***

^1^ Number of patients with at least one bacterium out of concomitant species. ^2^ n.s.—not statistically important difference when comparing *A. schaalii*-positive and -negative patients. * 0.01 < *p* ≤ 0.05; ** 0.001 < *p* ≤ 0.01; *** *p* ≤ 0.001.

## Data Availability

All datasets generated for this study are included in the manuscript and/or the supplementary files.

## Supplementary Materials

The following are available online at https://www.mdpi.com/2076-2607/9/3/669/s1, Figure S1: NMDS patients’ ordination, Figure S2: *A. schaalii* abundance and explanatory variables, Figure S3: Alpha diversity indices, Figure S4: Multivariable linear regression model fitted to species-based Richness, Figure S5: Multivariable linear regression model fitted to species-based Shannon´s index, Figure S6: Multivariable linear regression model fitted to species-based Simpson´s index, Figure S7: Multivariable linear regression model fitted to species-based Evenness, Figure S8: Analysis of similarities—ANOSIM, Table S1: Patients’ metadata, Table S2: Sample species matrix of joined culture an PCR-DGGE-S results, Table S3: Patient species matrix of joined culture an PCR-DGGE-S results, Table S4: NGS metadata, Table S5: Original species sample matrix, Table S6: Residual sample matrix, Table S7: Multiple regression model, Table S8: PERMANOVA on residual matrix, Table S9: RDA analysis on residual matrix.
