# Supplementary material for: Actinotignum schaalii: Relation to Concomitants and Connection to Patients’ Conditions in Polymicrobial Biofilms of Urinary Tract Catheters and Urines
Source: Microorganisms. 2021 Mar 23;9(3):669. doi: 10.3390/microorganisms9030669 (PMC8004716; doi:10.3390/microorganisms9030669)
Supplement: Supplementary file 1 [file microorganisms-09-00669-s001.zip › Supplementary_figures_proofread.pdf]

Supplementary Figures

Actinotignum schaalii: relation to concomitants and connection to patients' conditions in polymicrobial biofilms of urinary tract catheters and urines.

Iva Kotásková, Vít Syrovátka, Hana Obručová, Petra Vídeňská, Barbora Zwinsová, Veronika Holá, Eva Blašťíková, Filip Růžička, Tomáš Freiberger

Figure S1: NMDS patients' ordination

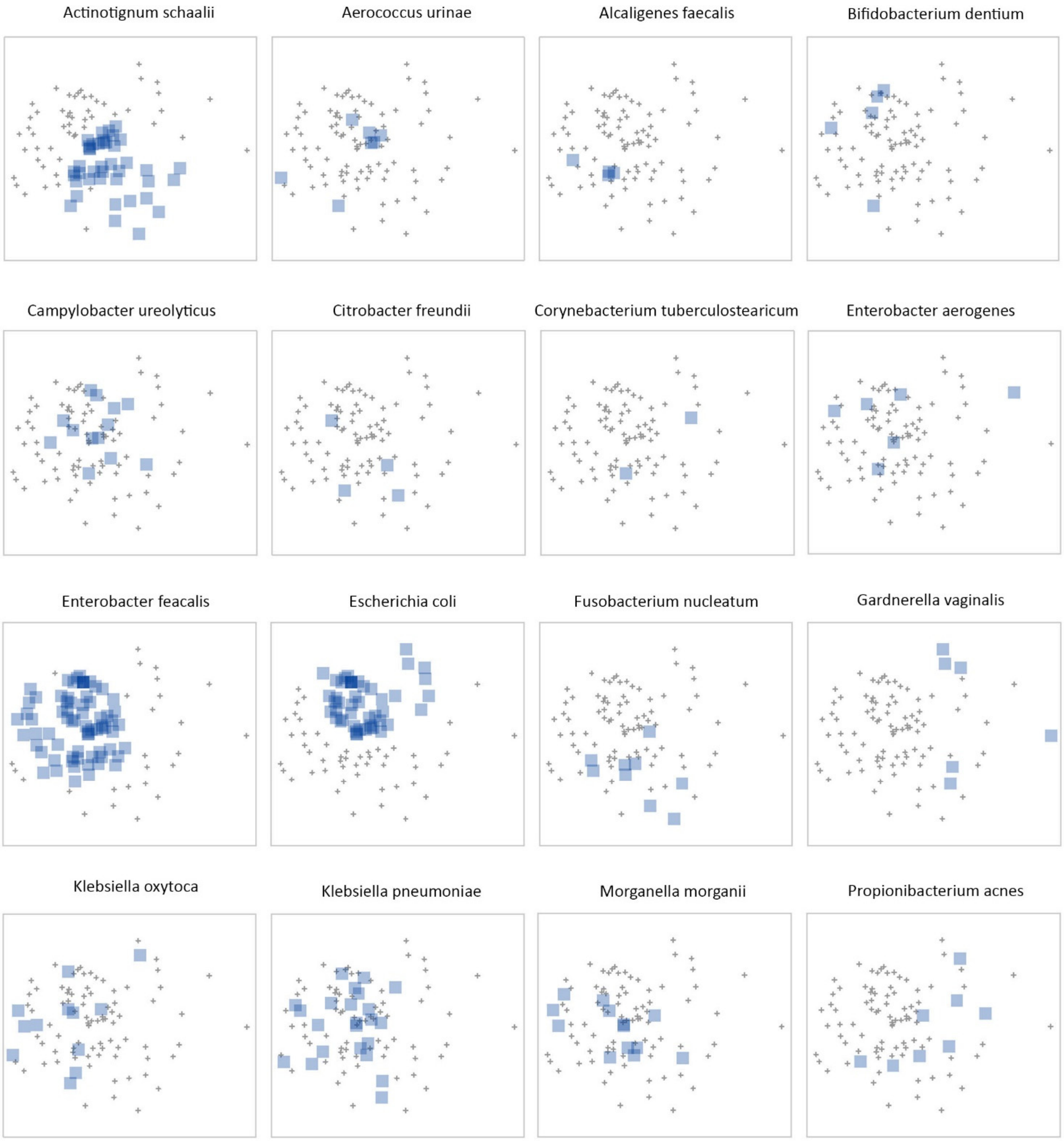

Figure S1 continues

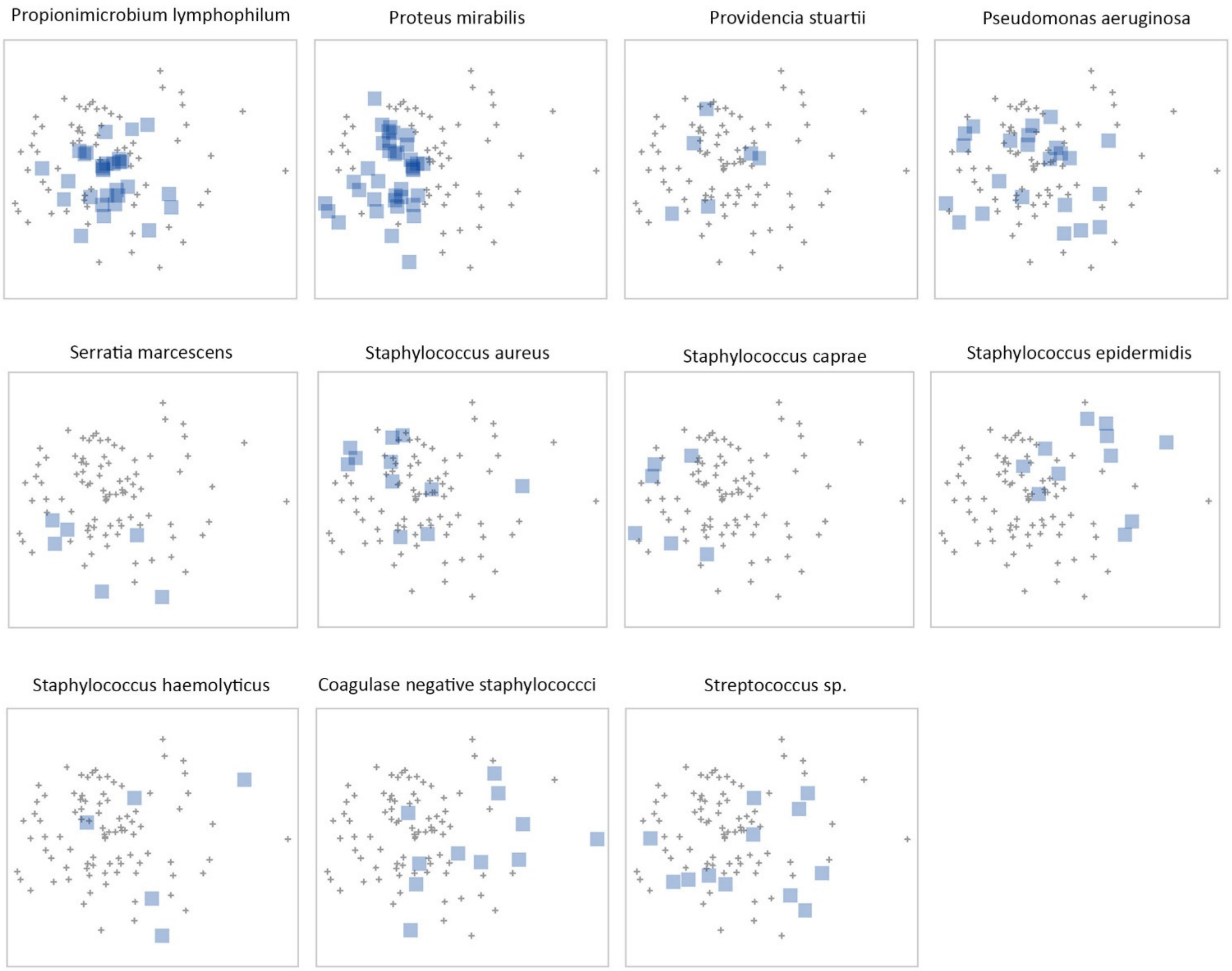

Plots show NMDS ordination of samples. Only patients with at least four representatives and those representatives with at least five occurrences were kept to calculate the ordination. This led to the elimination of extreme cases represented by rare species and patients with a low number of species. In each plot, the presence of bacteria in a sample is marked by the blue square. The taxonomy is indicated above each plot.

Figure S2: *A. schaalii* abundance and explanatory variables.

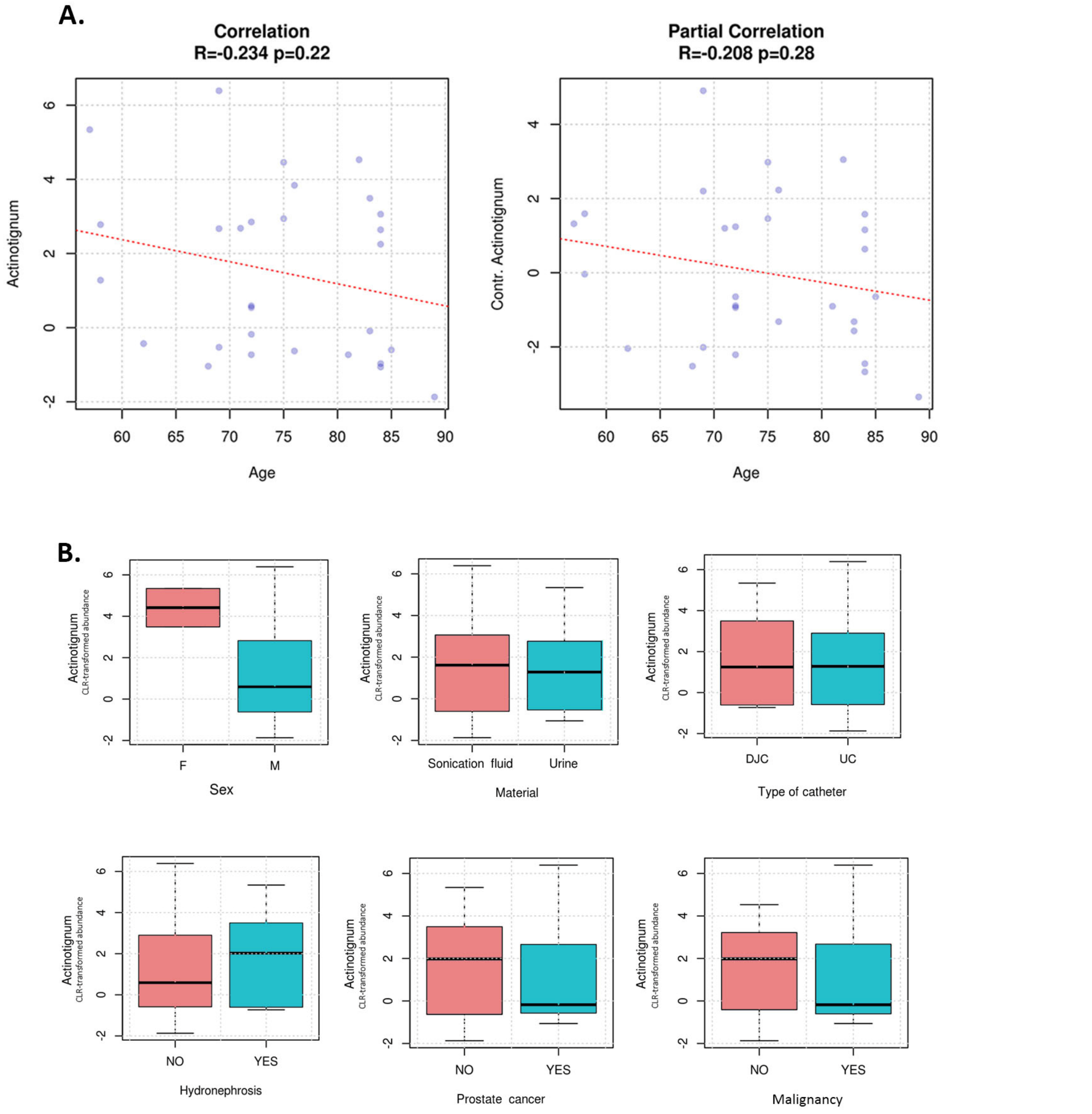

A. Scatterplots show the abundance of *Actinotigum* genus (CLR-transformed) plotted against the Age of patients. The Pearson correlation is given as R, the p-value indicates the significance of the correlation. In the Partial Correlation plot, *Actinotignum* abundance is controlled by the remaining factors entering the multiple regression (all other explanatory variables except *A. schaalii* presence/absence).

B. Boxplots show the association between all explanatory variables and abundance (CLR-transformed) of *Actinotignum* genus. *P*-values can be found in Supplement S1, Table S7, *Actinotignum* line.

Figure S3: Alpha diversity indices

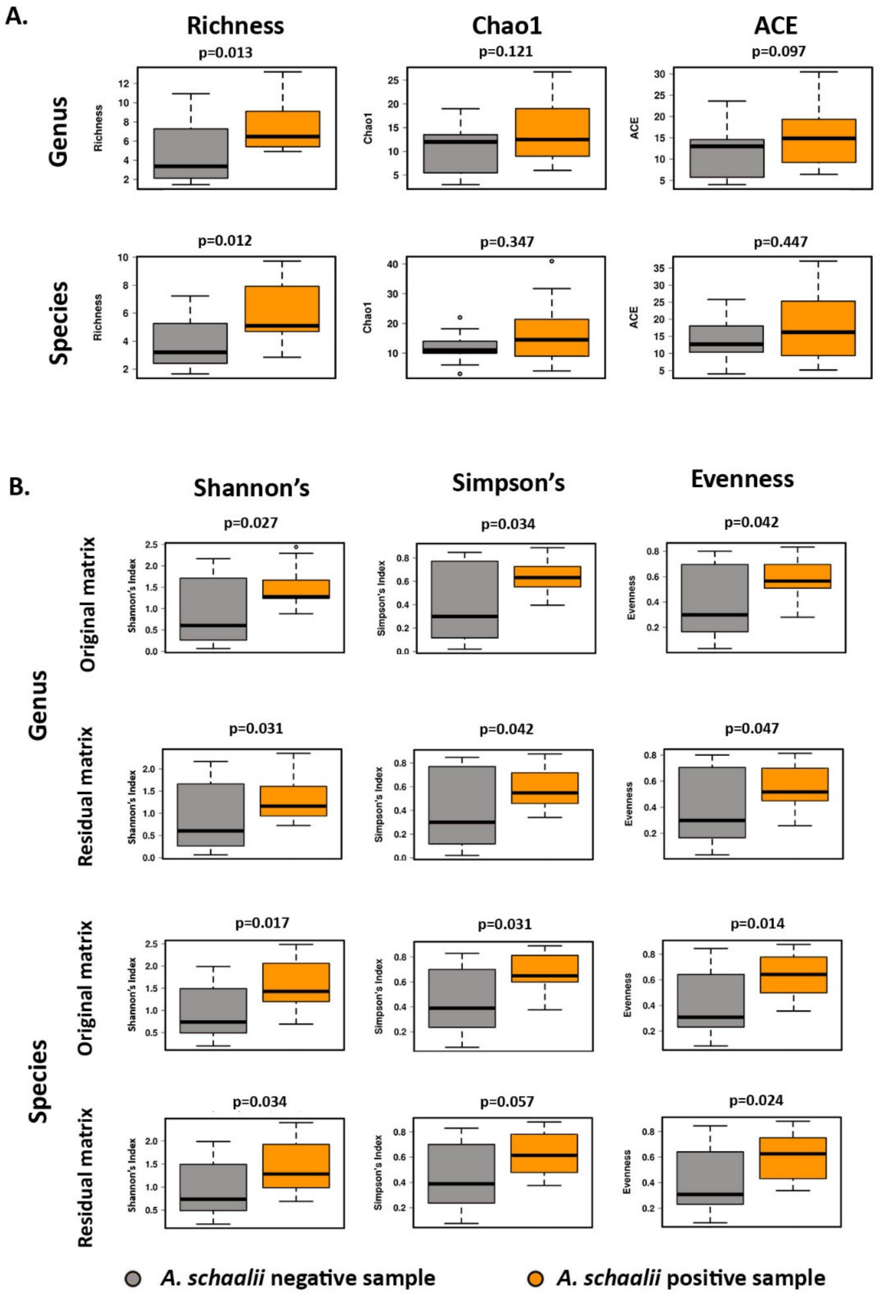

Figure shows boxplots of α-diversity indices in groups of *As-* (grey) and *As+* (orange) samples. Non-parametric Wilcoxon rank sum test was used to assess the statistical significance, and the p-value for each assessment is displayed above the boxplot.

A. Richness, Chao1 and ACE estimators are assessed at genus and species levels. Only genus and species Richness significantly differs between groups, indicating there is a difference in α-diversity, but not in singletons or doubletons (Chao1), or rare taxa (ACE).

B. Shannon´s and Simpson´s indices, and Evenness are assessed and compared for *As+* and *As-* samples. The absence of significant difference of the Simpson´s index derived from residual matrix referes that *A. schaalii* is one of dominant species in highly diverse *As+* samples.

Figure S4: Multivariable linear regression model fitted to species-based Richness.

Multivariable linear regression model:
Richness ~ *A. schaalii* presence + Age + Hydronephrosis + Prostate cancer + Malignancy + Sex + Material + Type of Catheter

	Coefficient	P
A.schaaliiYES	2.9499263	0.0105
Age	0.1132954	0.0819
HydronephrosisYES	0.4011591	0.8130
Prostate cancerYES	0.6697540	0.7930
MalignancyYES	-0.8493949	0.7280
SexM	-2.6513160	0.3690
MaterialUrine	1.0877094	0.3670
Type of catheterUC	2.7366946	0.1460

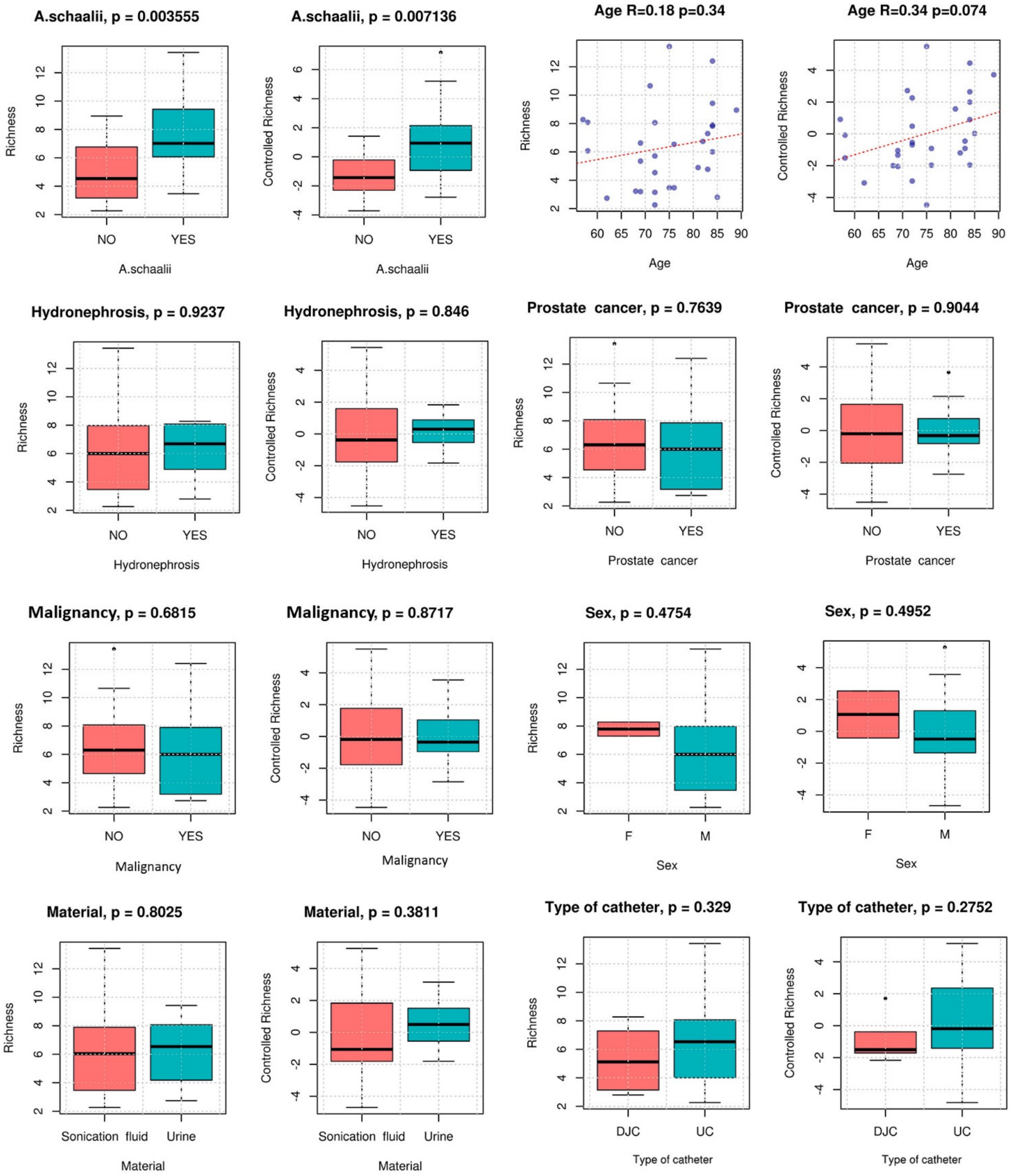

Figure S5: Multivariable linear regression model fitted to species-based Shannon´s index.

Multivariable linear regression model:
Shannon´s Index ~ *A. schaalii* presence + Age + Hydronephrosis + Prostate cancer + Malignancy + Sex + Material + Type of Catheter

	Coefficient	P
A.schaaliiYES	0.60573063	0.0117
Age	0.02175289	0.1080
HydronephrosisYES	0.24919189	0.4840
Prostate cancerYES	0.10575086	0.8420
MalignancyYES	-0.22069296	0.6660
SexM	-0.22632033	0.7110
MaterialUrine	0.20709083	0.4100
Type of catheterUC	0.43854669	0.2590

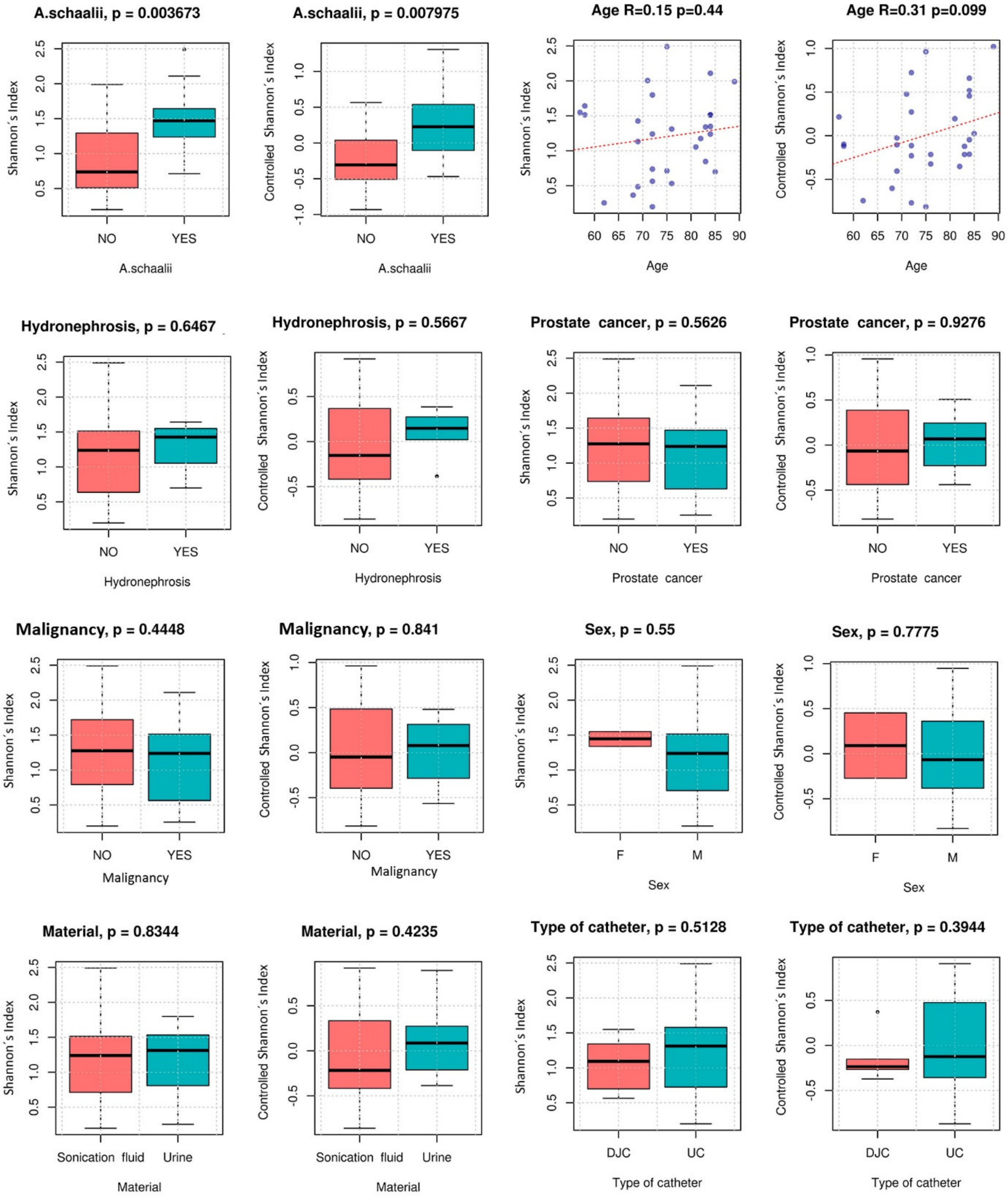

Figure S6: Multivariable linear regression model fitted to species-based Simpson´s index.

Multivariable linear regression model:
Simpson´s index ~ *A. schaalii* presence + Age + Hydronephrosis + Prostate cancer + Malignancy + Sex + Material + Type of Catheter

	Coefficient	P
A.schaaliiYES	0.245231382	0.00833
Age	0.008797699	0.09120
HydronephrosisYES	0.156779836	0.25700
Prostate cancerYES	0.049206437	0.81000
MalignancyYES	-0.094440024	0.63000
SexM	0.003791626	0.98700
MaterialUrine	0.082408200	0.39400
Type of catheterUC	0.130140239	0.38000

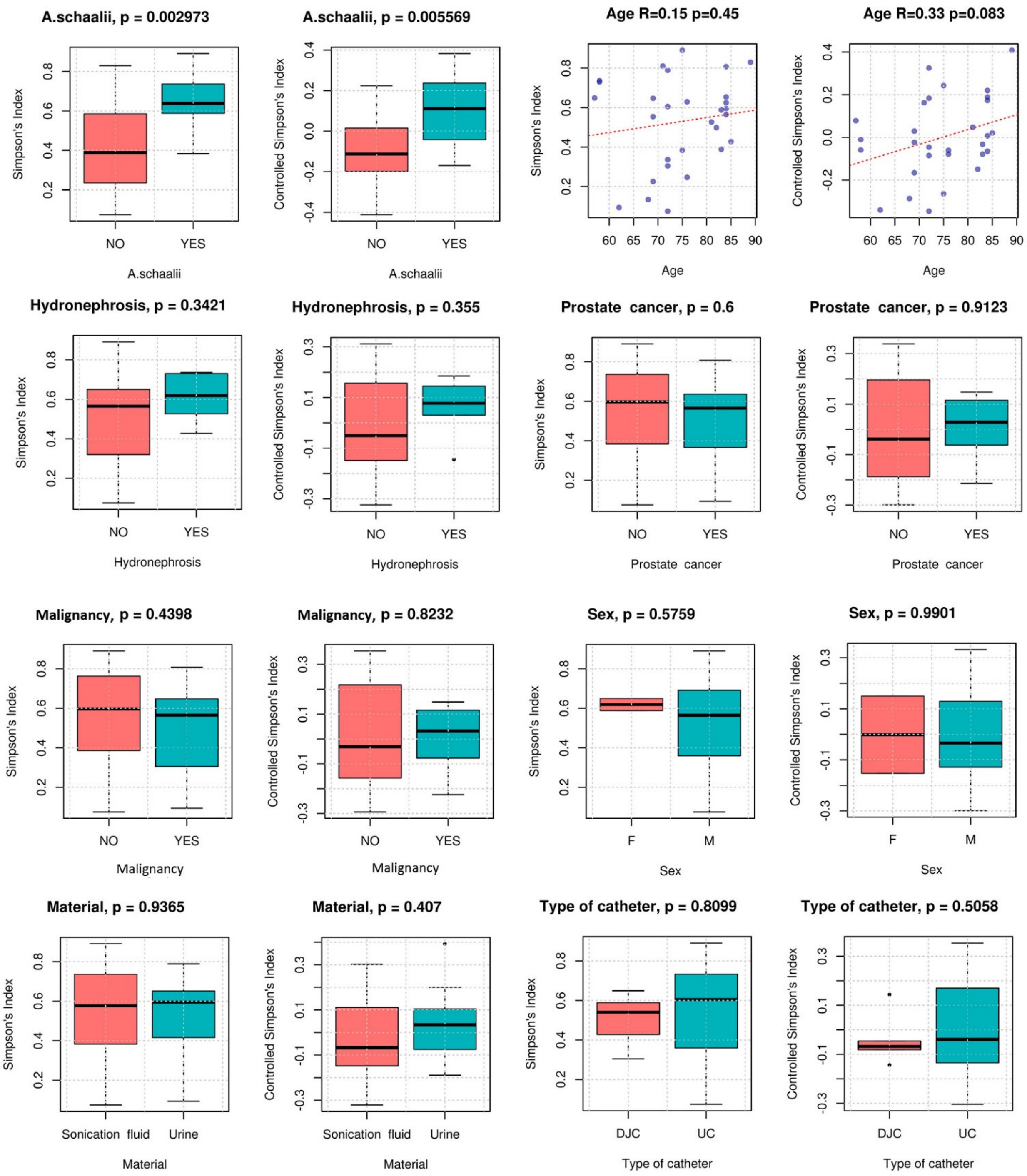

Figure S7: Multivariable linear regression model fitted to species-based Evenness.

Multivariable linear regression model:
Evenness ~ *A. schaalii* presence + Age + Hydronephrosis + Prostate cancer + Malignancy + Sex + Material + Type of Catheter

	Coefficient	P
A.schaaliiYES	0.219844806	0.00673
Age	0.005756549	0.19600
HydronephrosisYES	0.085261978	0.47300
Prostate cancerYES	0.157386662	0.37900
MalignancyYES	-0.146675376	0.39200
SexM	-0.007069751	0.97200
MaterialUrine	0.077246134	0.35800
Type of catheterUC	0.081814024	0.52300

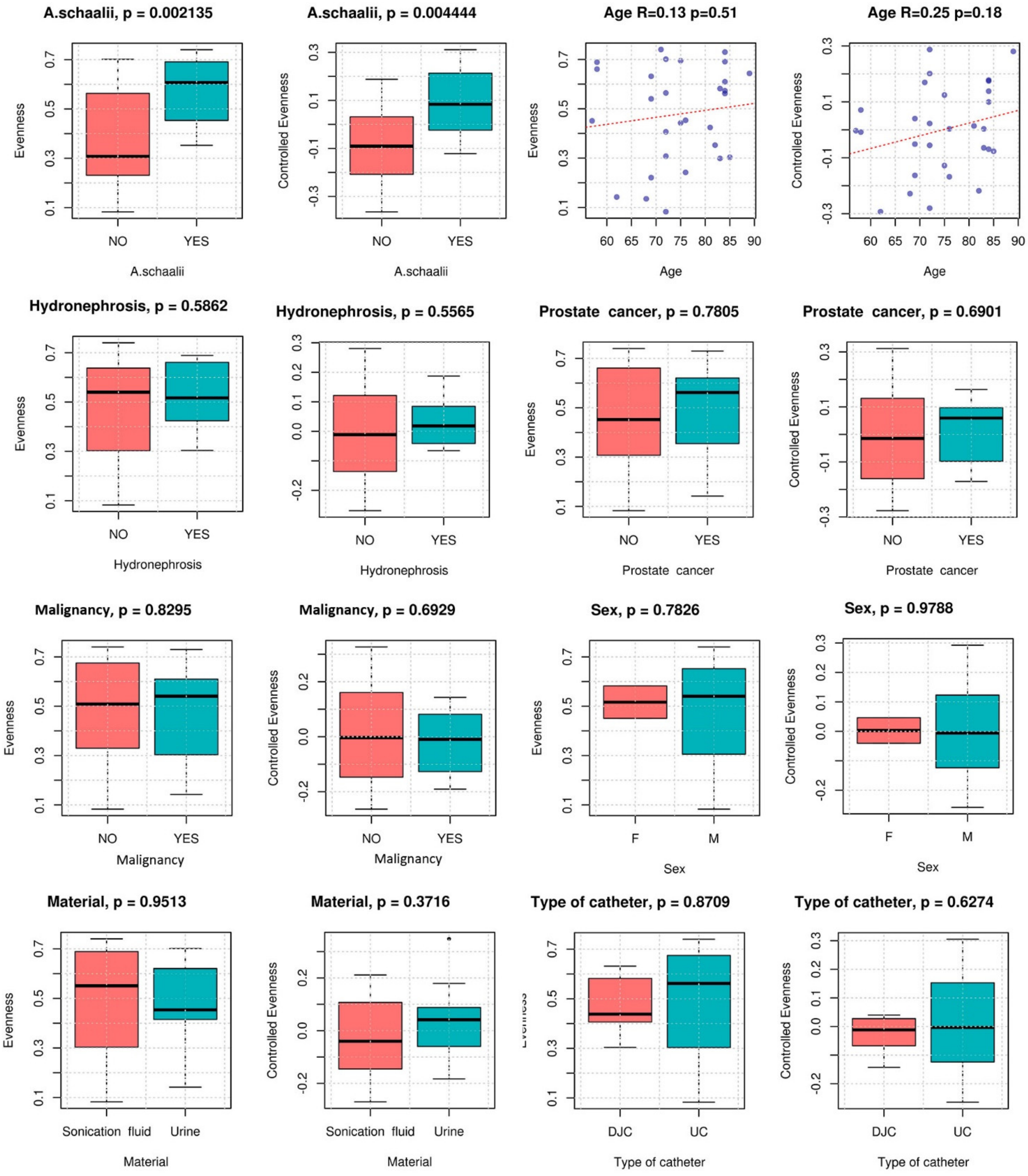

Figure S8: Analysis of similarities – ANOSIM.

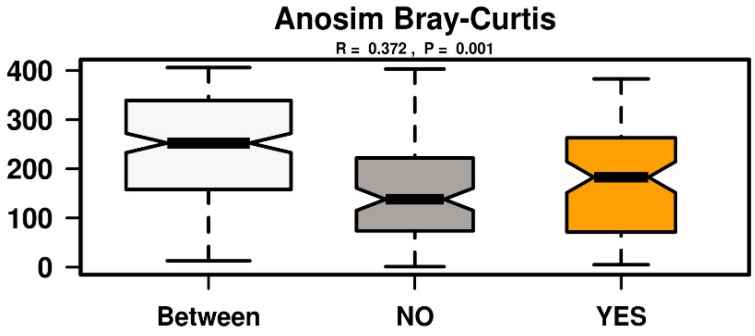

Anosim plot shows intra-group and between-group differences in community profiles (rank of values, y-axis) of As- samples (NO, grey) and As+ samples (YES, orange) expressed by the Bray-Curtis metric. The p-value is calculated by comparing intra-group and between-group distances. The plot shows the between-group variation is higher than intra-group variation in each of groups (As+ vs. As- samples).
